# Transient Response of Macroscopic Deformation of Magnetoactive Elastomeric Cylinders in Uniform Magnetic Fields

**DOI:** 10.3390/polym16050586

**Published:** 2024-02-21

**Authors:** Gašper Glavan, Inna A. Belyaeva, Mikhail Shamonin

**Affiliations:** East Bavarian Centre for Intelligent Materials (EBACIM), Ostbayerische Technische Hochschule (OTH) Regensburg, Seybothstr. 2, 93053 Regensburg, Germany; inna.belyaeva@oth-regensburg.de (I.A.B.); mikhail.chamonine@oth-regensburg.de (M.S.)

**Keywords:** magnetoactive elastomer, magnetorheological elastomer, macroscopic deformation, magnetostriction, time-varying magnetic field, time behaviour

## Abstract

Significant deformations of bodies made from compliant magnetoactive elastomers (MAE) in magnetic fields make these materials promising for applications in magnetically controlled actuators for soft robotics. Reported experimental research in this context was devoted to the behaviour in the quasi-static magnetic field, but the transient dynamics are of great practical importance. This paper presents an experimental study of the transient response of apparent longitudinal and transverse strains of a family of isotropic and anisotropic MAE cylinders with six different aspect ratios in time-varying uniform magnetic fields. The time dependence of the magnetic field has a trapezoidal form, where the rate of both legs is varied between 52 and 757 kA/(s·m) and the maximum magnetic field takes three values between 153 and 505 kA/m. It is proposed to introduce four characteristic times: two for the delay of the transient response during increasing and decreasing magnetic field, as well as two for rise and fall times. To facilitate the comparison between different magnetic field rates, these characteristic times are further normalized on the rise time of the magnetic field ramp. The dependence of the normalized characteristic times on the aspect ratio, the magnetic field slew rate, maximum magnetic field values, initial internal structure (isotropic versus anisotropic specimens) and weight fraction of the soft-magnetic filler are obtained and discussed in detail. The normalized magnetostrictive hysteresis loop is introduced, and used to explain why the normalized delay times vary with changing experimental parameters.

## 1. Introduction

In recent years, there is growing interest in the investigation of magnetic-field-induced macroscopic deformations of an intriguing class of polymer-based ferromagnetic composite materials, known as magnetorheological or magnetoactive elastomers (MAEs) [1,2,3,4,5,6,7,8,9,10,11,12,13]. In general, MAE comprise micro- or nanometer-sized ferromagnetic particles embedded into a soft elastomer matrix [14,15,16,17,18,19,20,21]. The reason for this surge of interest is determined by much higher deformations of MAEs (longitudinal strain up to 10^−2^–10^−1^) in technically feasible magnetic fields (few hundred kA/m) in comparison to conventional (pure metals and alloys) magnetostrictive materials (strain up to about $2\times 10^{-3}$) [22], which makes them promising for potential applications as magnetically controlled soft actuators in soft robotics [23,24] and magnetic field sensors [10]. The change in the shape or the dimensions of a solid magnetic material induced by a change in its magnetic state is commonly designated as magnetostriction [25]. This term is also used in the literature with respect to MAEs, although the physical origin of macroscopic deformation in mechanically soft (shear modulus of the matrix is below 10 kPa) MAEs [2,20,26] is different from that in conventional ferromagnetic crystals, where it is mainly due to spin–orbit coupling [27]. In the definition of magnetostriction, it is also implied that the applied magnetic field causing the changes is uniform.

Very recently, several aspects of magnetostrictive behaviour in MAEs came into the focus of different research groups, leading to significant progress in enhancement of strains in MAEs. Silva at al. [8] used helicoidal-shaped particles from a Fe-Co alloy embedded into a soft elastomer matrix to achieve a giant strain of 3731 ppm at an unprecedently low volume fraction of particles of just 3.4 vol%. Tasin et al. [10] studied the MAEs of the moderate stiffness (shear storage modulus above 300 kPa) and reached the maximum strain of ≈7.5 × $10^{-4}$ in a highly filled MAE with 80 wt% of iron (Fe). Glavan et al. [11] investigated soft MAE cylinders and achieved the highest reported strain of ≈0.35 for an MAE sample with 75 wt% of iron and an aspect ratio of 0.2. There are simultaneous lines of research on magnetostriction in MAEs, which originate from different constraints on material parameters (material stiffness or particle concentration). The present paper concerns mechanically soft MAE cylinders, previously investigated in the quasi-static case in Ref. [11]. These samples comprise µm-sized iron particles dispersed in a soft elastomer matrix (shear storage modulus ≈7.7 kPa).

Hitherto, most publications on macroscopic deformation of MAE samples in uniform magnetic fields were devoted to the quasi-static behaviour, where the deformation has reached its stationary state after the magnetic field has been changed. However, it is known that the rheological response of MAE material to a time varying magnetic field results in complex transient behaviour, which may be characterized by several time constants [28,29]. The rheological relaxation behaviour of magnetic gels after magnetic field alternation was recently considered in [30,31]. The transient response of the MAE permittivity to a step magnetic-field excitation can be rather complex as well [32]. Very recently, Kubík et al. [33] investigated the transient response of the field-induced force of constrained MAE cylinders to the step-like magnetic-field excitation and found that the force dynamics can be characterized as a first-order system with the time constant in the 27–120 ms range. However, direct measurements of the dynamics of macroscopic deformation of MAE samples in time varying magnetic fields are still missing in the literature. Such an experimental information is of particular interest because understanding the dynamics of nonlinear soft actuators is crucial to creating controllable soft robots [34,35].

The purpose of this paper is to systematically investigate the transient behaviour of macroscopic deformation of MAE cylinders to trapezoidal field excitations with the slew rate of magnetic field between ≈52 kA/(s·m) and ≈757 kA/(s·m).

The paper is organized as follows: Section 2 briefly recalls the fabrication of MAE cylinders and explains the experimental setup. The measurement protocol is described in detail. The experimental results on the transient response are presented and discussed in Section 3 for different slew rates of the magnetic field and various field amplitudes. Conclusions are drawn in the final Section 4.

## 2. Materials and Methods

### 2.1. MAE Cylinders

All MAE materials were based on the same polydimethylsiloxane (PDMS) matrix obtained at the stoichiometry ratio of the reaction r≈1, where *r* is the ratio of the molar concentrations of hydride and vinyl reactive groups. In the ideal case, there should be only elastically active chains in the polymer network [36]. The entire procedure of MAE synthesis was already described in detail in our previous works [32,37]. The magnetic filler was a carbonyl iron powder (CIP; type SQ, BASF SE Carbonyl Iron Powder and Metal Systems, Ludwigshafen, Germany) with the mean particle diameter of 3.9–5.0 µm. An initial compound was mixed together with the CIP particles and a crosslinker. The crosslinking reaction was activated by a Pt catalyst. For the control of the Pt catalyst’s activity, an inhibitor was employed. The 3D printed thermoresistant moulds from acrylonitrile butadiene styrene (ABS) were filled with a finished, but uncured MAE composition. The air bubbles in the not yet cured MAE specimens were removed using a vacuum desiccator for about 7 min [37].

The cylinders differed in the mass fraction of CIP (70, 75 and 80 wt%, corresponding to approximately 22, 27 and 33 vol%), anisotropy of internal particle arrangement (denoted as isotropic/anisotropic) and the aspect ratio $\Gamma_{0}=h_{0}/d_{0}$ (0.2, 0.4, 0.6, 0.8, 1.0 and 1.2), where the initial diameter $d_{0}$ was kept constant at 15 mm and the initial cylinder height $h_{0}$ was varied. Isotropic cylinders were cured in the absence of a magnetic field. To obtain anisotropic cylinders, two samarium cobalt (SmCo) permanent magnets (diameter 25 mm, thickness 5 mm, magnetization along thickness) were placed in filled moulds. The entire assemblies were left for about one minute, before they were put in an oven to cure. The distance between magnets was kept constant at 22 mm, so that magnetic field in the middle between magnets was approximately 95 kA/m [11].

For each material composition, reference samples were produced to analyse rheological properties of MAE materials synthesized. Rheological properties were analysed with a commercial rheometer (Anton Paar, model Physica MCR 301, with a magnetorheological device (MRD301) and the plate-plate PP20/MRD/TI geometry) at a fixed angular frequency ω=10 s^−1^ and a shear oscillation amplitude γ=0.01%. The results of rheological measurements were given in [11].

### 2.2. Experimental Setup

**Figure 1:**
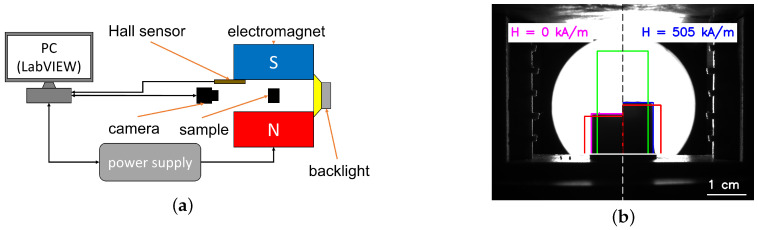
(**a**) Schematic diagram of the experimental setup. (**b**) Comparison of experimental images in the absence and in the presence of a magnetic field. The left side of the combined image presents a half of an isotropic cylinder with 75 wt% of iron and an aspect ratio $\Gamma_{0}=0.6$ in zero magnetic field, and the right side shows a half of the same cylinder in a magnetic field H=505 kA/m. The regions of interest (ROIs) for obtaining the cylinder’s height and width are designated by green and red lines, respectively. The cylinder base plane is shown by the white line. The contours of non-deformed and deformed cylinders are drawn by magenta and blue lines, correspondingly.

Figure 1 schematically shows the experimental setup used. MAE cylinders were glued onto a 3D printed holder from polylactic acid (PLA) and placed between the poles of an electromagnet (EM2 model, MAGMESS Magnetmesstechnik Jürgen Ballanyi e.K., Bochum, Germany), powered by a bi-polar power supply (FAST-PS 1k5, CAENels s.r.l., Basovizza, Italy). The generated magnetic field is known to be highly uniform [38]. The deformation of cylinders was captured in sideview using a CMOS camera (Alvium 1800 U-319 m, Allied Vision Technologies GmbH, Stadtroda, Germany) with a suitable lens (Edmund Optic Double Gauss Focusable, 25 mm C-mount F4.0 1.300, Barrington, NJ, USA). Cylinders were backlight illuminated with a light emitting diode (LED; Illuminant G4 Pen, Conrad Electronics, Hirschau, Germany) through a diffuser (Perspex diffuse, 2.5 mm, 3A Composites GmbH, Sins, Switzerland). Cylindric samples were positioned vertically between the poles of the electromagnet in such a way that the cylinder axis was parallel to magnetic field lines. The vertical position of the camera was adjusted in such a way that the angle between camera and top edge of the cylinder remained constant. The experiment was automatized using LabVIEW software (version 2018, National Instruments, Austin, TX, USA) [11].

### 2.3. Image Processing

From images, the variations in diameter and height of the outer contour of an MAE cylinder were obtained using simple thresholding, followed by the Cunny edge detection algorithm in the OpenCV library written in Python (Figure 1b). The baseline of a cylinder was determined manually and fixed for subsequent analysis. The rectangular ROI (green lines in Figure 1b) had a width of 400 px and served to determine the cylinder height. The size of one pixel in the image plane was about 28.4 µm × 28.4 µm. There were two separate ROIs (red lines in Figure 1b) for the two vertical sides of a cylinder. The height *h* was calculated as a distance between the top edge (calculated as an average of vertical (*y*) coordinates of the edge pixels inside of the green rectangle) and the fixed baseline. The red ROIs were adjusted according to the cylinder deformation in such a way that their horizontal edges were 20 px below the average cylinder height. The left and right vertical cylinder edges were determined separately as an average of horizontal (*x*) coordinates of edge pixels inside the red rectangles. The cylinder width *d* was then calculated as the distance between the left and right cylinder edges. The magenta and red rectangles in Figure 1b have the dimensions obtained with the above algorithm. Apparent longitudinal $\lambda_{\|}=\left(h-h_{0}\right)/h_{0}$ and transversal $\lambda_{⊥}=\left(d-d_{0}\right)/d_{0}$ strains were calculated from the observed changes in height *h* and diameter *d*.

### 2.4. Measurement Protocol

**Figure 2:**
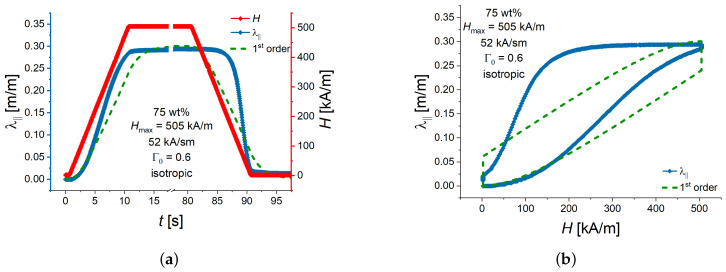
(**a**) Example of the transient response of the longitudinal strain $\lambda_{\|}$ to a single trapezoidal pulse of the external magnetic field. (**b**) The same data presented as the field dependence of the longitudinal strain. The green dashed line shows the best fit to the time dependence of the longitudinal strain assuming the transfer function of the first order. The MATLAB system identification toolbox was used.

The driving current *I* in the electromagnet’s coils was varied between 0 and 10 A, which corresponded to the magnetic field range between 0 and 505 kA/m. The specific feature of the power supply to the electromagnet was that it allowed one to set up the slew rate of the driving current (see Figure 2) in the coils of the electromagnet, so that the resulting ramp was practically linear function of time (see Figure 2a). The highest possible slew rate in our experiments was 15 A/s, corresponding to ≈757 kA/(s·m). Higher slew rates could not be achieved due to the internal protection algorithm of the power source, which switches it off, when the driving current is diminished too fast. In this case, the inductance of the electromagnet behaves itself like a power source, which must be compensated by the electronic circuitry. Table 1 summarizes the investigated slew rates of the driving current *I* and the resulting external magnetic field *H*. Another limitation is the maximum image acquisition rate of the camera of 20 fps corresponding to the sampling interval of 50 ms. For the highest slew rate of the electrical current and the lowest current amplitude $I_{max}=3$ A, the camera could provide four images when the electrical current was either increasing or decreasing.

The current *I* was first linearly increased with time up to the maximum value $I_{max}$ at a given slew rate, kept constant for at least 20 s, so that the steady state deformation of the cylinder could be reached, and finally, the current was decreased to zero at the same slew rate. The images of the cylinder were taken at a frame rate of 20 fps. The maximum currents and the corresponding maximum magnetic field strengths are summarized in Table 2.

Horváth and Szalai [39] studied the time-domain magnetic susceptibility response to ramp excitation in the weak-field limit (H<10 kA/m), which was found to be well approximated with the response of a first-order system to ramp excitation. In the steady state region, the normalized response lagged behind the normalized ideal excitation by the time delay, which was equal to the sum of the lag of the excitation and the response time of the MRE. Such an approach did not work in our case, because we could not observe the parallelism of the strain response to the ramp excitation in the time domain.

Kubík et al. [33,40] used the MATLAB system identification toolbox to calculate the parameters of the (delayed) first-order transfer function for the transient response of a MAE and a magnetorheological fluid and to deduce the resulting time constants. In [33], it was noted that the assumption of a linear dynamic system is just a simplification. Because the magnetostriction phenomenon is known to be non-linear and hysteretic with respect to applied magnetic field (see, e.g., [41,42]), an approach using linear transfer functions to determine time constants of the macroscopic deformation of MAEs in sufficiently high magnetic fields may not work satisfactory. As an example, Figure 2 shows calculated response of the longitudinal elongation $\lambda_{\|}$ to an applied magnetic field *H*, assuming the linear system of the first order in the MATLAB system identification toolbox. It is seen that the agreement between calculated and experimental values of $\lambda_{\|}$ is poor. Moreover, a careful visual inspection of the experimental transient response of $\lambda_{\|}$ reveals that its behaviour in ascending and descending magnetic fields is somewhat different.

**Figure 3:**
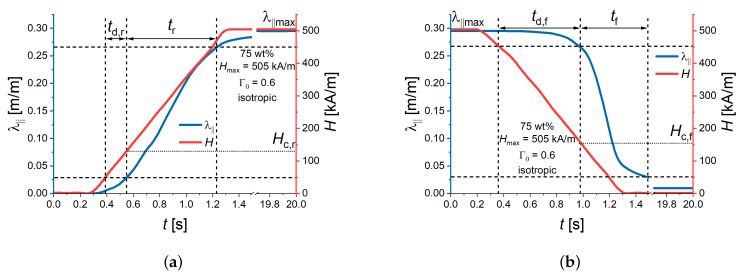
Example of the transient response of the longitudinal strain $\lambda_{\|}$ for $H_{max}=505$ kA/m and a magnetic field rate of 505 kA/(s·m) for an isotropic MAE cylinder with 75 wt% of CIP and an aspect ratio $\Gamma_{0}=h_{0}/d_{0}=0.6$ (**a**) Definition of delay rise time $t_{d,r}$ and rise time $t_{r}$. (**b**) Definition of delay fall time $t_{d,f}$ and fall time $t_{f}$. $\lambda_{\|max}$ denotes the maximum strain value in this experiment. $H_{c,r}$ and $H_{c,f}$ denote the corresponding critical magnetic fields.

Therefore, a more robust, empirical determination of characteristic times was employed. Figure 3 illustrates the definitions of the delay times $t_{d,r}$, $t_{d,f}$ of the deformation for the rising (increasing) and falling (decreasing) parts of the external magnetic field, respectively. Further, the rise time $t_{r}$ and the fall time $t_{f}$ were determined from the recorded values of the magnetic field and the cylinder deformation. Definitions of delay, rise and fall times are given in Equation (1) for the ascending field and in Equation (2) for the descending part of the external magnetic field.

(1) $$\begin{array}{cc} t_{d,r} & =t\left(\lambda =0.1\cdot \lambda_{max}\right)-t\left(H=0.1\cdot H_{max}\right),\\ t_{r} & =t\left(\lambda =0.9\cdot \lambda_{max}\right)-t\left(\lambda =0.1\cdot \lambda_{max}\right), \end{array}$$

(2) $$\begin{array}{cc} t_{d,f} & =t\left(\lambda =0.9\cdot \lambda_{max}\right)-t\left(H=0.9\cdot H_{max}\right),\\ t_{f} & =t\left(\lambda =0.1\cdot \lambda_{max}\right)-t\left(\lambda =0.9\cdot \lambda_{max}\right). \end{array}$$

The rise time is synonymous with the transition duration of a positive-going transition, and the fall time is synonymous with the transition duration of a negative-going transition [43]. The 10% (0.1) and 90% (0.9) reference levels are commonly used values in electrical and electronic engineering, see, e.g., [35,43,44].

Additionally, we defined the critical magnetic field values $H_{c,r}$, $H_{c,f}$ at which the strain commences to change significantly during the transient processes. $H_{c,r}$ was determined at the same time point as $t_{d,r}$, and $H_{c,f}$ was calculated at the same time point as $t_{d,f}$ (Figure 3).

## 3. Results and Discussion

### 3.1. Slew Rate Dependence

Firstly, the characteristic times were compared for different slew rates of driving magnetic field: 52, 254, 505 and 757 kA/(s·m). The maximum value of magnetic field $H_{max}$ was kept constant at 505 kA/m.

**Figure 4:**
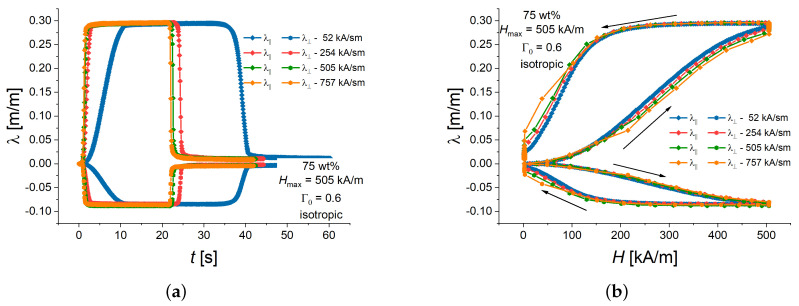
Example of longitudinal and transversal strain response to different magnetic field slew rates for isotropic MAE cylinders with 75 wt% and $\Gamma_{0}=0.6$ in dependence on time (**a**) and the same experimental results in dependence on the magnetic field (**b**). The arrows denote the direction of changes in strains and magnetic field with time. The error bars are omitted for the sake of clarity.

As an example, Figure 4a shows longitudinal and transversal strain responses of an isotropic MAE cylinder with 75 wt% of iron particles and an aspect ratio $\Gamma_{0}$ of 0.6 plotted versus the time. Here, and in the following figures, the lines connecting experimental points serve as a guide to the eye. Figure 4b presents that same data plotted against the external magnetic field. It can be seen from Figure 4b that the strain hysteresis curves were rather similar for all slew rates of applied magnetic field. A careful inspection of the curves in Figure 4b reveals that the stain at a particular value of the applied magnetic field at the same momentary value of the applied magnetic field was lower for a higher slew rate in comparison with a lower slew rate. This can be seen well for the highest slew rate in the maximum magnetic field of 505 kA/m, where the creep phenomenon was obvious, i.e., there was continued deformation of a viscoelastic material after the magnetic load has reached a constant state. In the maximum magnetic field, the experimental longitudinal-strain values corresponding to subsequent time steps increased as the strain reached the steady state value. However, the steady state value of both longitudinal and transverse strains seemed to be independent of the slew rate in the investigated range of rates.

To clarify how the characteristic times were related to the actual slew rate, they were normalized on the rise (or fall) time of the applied magnetic-field ramp $t_{ramp}$. $t_{ramp}$ was defined as the time taken by the driving current to rise from 10% to 90% of its final value. The normalized characteristic times are denoted with the tilde ($\tilde{t}$) in the following.

Our definitions of the critical magnetic-field values are referred to the maximum strain values reached in a maximum magnetic field $H_{max}$. Therefore, comparison of the absolute values of $H_{c,r}$, $H_{c,f}$ should not be performed for different values of $H_{max}$. If the magnetic field increases (or decreases) linearly at a constant rate between zero and $H_{max}$ ($H_{max}$ and zero, respectively), it can be easily shown (Appendix A) that:

(3) $$0.1+0.8{\tilde{t}}_{d,r}=\frac{H_{c,r}}{H_{max}}$$

and

(4) $$0.9-0.8{\tilde{t}}_{d,f}=\frac{H_{c,f}}{H_{max}}.$$

Note that, for the power-down process, 10% of the maximum strain can be achieved after the magnetic field already vanished (Figure 3b). In this case, $\left({\tilde{t}}_{d,f}+{\tilde{t}}_{f}\right)>0.9/0.8=1.125$. Similarly, for the power-up process, 90% of the maximum strain can be reached after the magnetic field attained its maximum value, resulting in $\left({\tilde{t}}_{d,r}+{\tilde{t}}_{r}\right)>1.125$.

**Figure 5:**
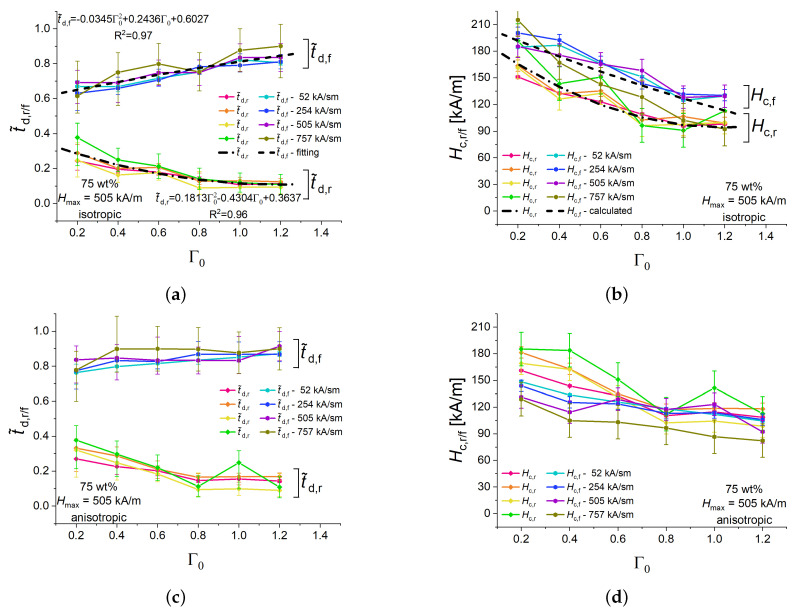
Dependencies of the normalized rise and fall delay times of isotropic cylinders with 75 wt% of iron particles for different magnetic field ramps (**a**) and the corresponding values of critical magnetic fields (**b**). Dependencies of the normalized delay times of anisotropic cylinders for different magnetic field ramps (**c**), and the corresponding values of the critical magnetic field (**d**).

Figure 5 presents the dependencies of the normalized delay times ${\tilde{t}}_{d,r}$, ${\tilde{t}}_{d,f}$ and the corresponding critical magnetic fields $H_{c,r}$, $H_{c,f}$ on the aspect ratio $\Gamma_{0}$ isotropic and anisotropic cylinders with 75 wt% of Fe for a fixed peak value $H_{max}=505$ kA/m and different magnetic field slew rates. The experimental points in Figure 5 refer to the longitudinal strain. It was observed that the normalized delay times for power-up were lower than the normalized delay times for power-down. The differences between normalized delay times of a particular transient process (power-up or power-down) for investigated slew rates were minor, within the uncertainty of measurements (Figure 5a). Therefore, it is reasonable to assume that the critical magnetic fields for a particular transient process did not depend on the slew rate for the same aspect ratio $\Gamma_{0}$ (Figure 5b). The dashed and dash-dotted curves in Figure 5a represent fitted second-order polynomials for the dependencies of both families of normalized delay times ${\tilde{t}}_{d,r}$, ${\tilde{t}}_{d,f}$ on the aspect ratio $\Gamma_{0}$, respectively. For a given $\Gamma_{0}$, the mean value of a specific characteristic time (${\tilde{t}}_{d,r}$ or ${\tilde{t}}_{d,f}$) was calculated with the same weight for all experimental points. The polynomials were fitted to these mean values. $R^{2}$ denotes a determination coefficient. These dependencies were re-calculated into the critical fields $H_{c,r}$, $H_{c,f}$ using Equations (3) and (4). The resulting dependencies of the critical fields $H_{c,r}$, $H_{c,f}$ on the aspect ratio $\Gamma_{0}$ are shown in Figure 5b by dashed and dash-dotted lines, respectively. A good agreement with the experimentally determined values of the critical fields was observed there.

The critical magnetic field $H_{c,r}$ for the power-on process was lower than the critical magnetic field $H_{c,f}$ for the power-down process at a fixed aspect ratio $\Gamma_{0}$, consistent with Figure 5a. In general, the restructuring of particles in an increasing magnetic field needed less time than their restructuring in decreasing magnetic field, which is obvious from ${\tilde{t}}_{d,r}<{\tilde{t}}_{d,f}$ in Figure 5a. Moreover, it turned out that, in general, $\left({\tilde{t}}_{d,r}+{\tilde{t}}_{r}\right)≲\left({\tilde{t}}_{d,f}+{\tilde{t}}_{f}\right)$ for the same set of experimental parameters. The corresponding curves for the sums of normalized characteristic time constants $\left({\tilde{t}}_{d,r}+{\tilde{t}}_{r}\right)$, $\left({\tilde{t}}_{d,f}+{\tilde{t}}_{f}\right)$, expressing the total duration of transient processes, are given in Appendix B for all experimental results in the present paper.

Remanent strains in zero magnetic field and their relaxation to the undeformed state are clearly visible in Figure 4b. This can be attributed to the hysteresis of the consolidation of filler particles into elongated aggregates, i.e., dependence of the internal microstructure of particles on the magnetization history [45,46]. In recent in situ observations of particle rearrangements in polyurethane-based MAEs, most of the aggregates did not return to the original position even 5 min after the magnetic field was switched off [47].

The field $H_{c,r}$ can be interpreted as the external magnetic field at which the magnetic interactions commence to overcome the elastic interactions between magnetized particles for a linearly increasing external magnetic field, while the field $H_{c,f}$ can be interpreted as the external magnetic field at which the magnetic interactions begin to succumb the elastic interactions between particles for a linearly decreasing external magnetic field. Both critical fields decrease with the increasing aspect ratio $\Gamma_{0}$, which can be attributed to the decreasing demagnetizing field in specimens with increasing aspect ratio. Correspondingly, ${\tilde{t}}_{d,r}$ decreased and ${\tilde{t}}_{d,f}$ increased with increasing $\Gamma_{0}$.

Pre-structured (anisotropic) samples showed qualitatively similar dependences of the normalized delay times ${\tilde{t}}_{d,r}$ and ${\tilde{t}}_{d,f}$ the critical magnetic fields $H_{c,r}$, $H_{c,f}$ on the aspect ratio $\Gamma_{0}$ (Figure 5c,d). The delay times ${\tilde{t}}_{d,r}$ for the power-up processes were similar for isotropic and anisotropic samples. For the power-down processes, ${\tilde{t}}_{d,f}$ were about 0.1 higher for anisotropic samples than for their isotropic counterparts (Figure 5a,c) and the differences between the $H_{c,r}$– and $H_{c,f}$– families of curves were not pronounced as well as they were for isotropic samples (Figure 5b,d). This is mostly due to the lower values of $H_{c,f}$ for anisotropic samples in comparison with isotropic samples. A possible explanation is that the reduction of the external magnetic field can destroy the elongated structures in non-structured samples easier than in pre-structured samples.

**Figure 6:**
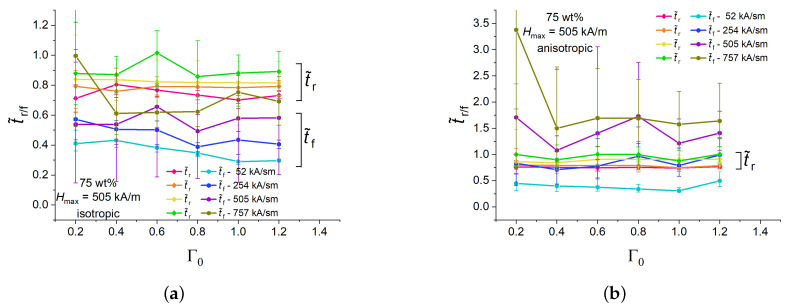
Dependencies of the normalized rise and fall times of isotropic (**a**) and anisotropic (**b**) MAE cylinders with 75 wt% of iron on the aspect ratio $\Gamma_{0}$ for different magnetic field ramps and fixed $H_{max}=505$ kA/m.

Figure 6 depicts the dependences of the normalized rise and fall times ${\tilde{t}}_{r}$, ${\tilde{t}}_{f}$ on the aspect ratio $\Gamma_{0}$ for isotropic and anisotropic cylinders with 75 wt% of Fe for a fixed peak value $H_{max}=505$ kA/m and different magnetic field slew rates. The experimental points in Figure 6 refer to the longitudinal strain. For a given slew rate in isotropic samples, normalized rise times are higher than fall times (Figure 6a) and seemingly independent of the aspect ratio $\Gamma_{0}$. Both normalized rise and fall times increase with increasing slew rates. This can be attributed to the retardation of the restructuring (movement) of filling particles in a viscoelastic medium with respect to the fast-changing magnetic field. The normalized rise times of anisotropic samples exhibit similar numerical values and dependencies as their isotropic counterparts (Figure 6b). However, normalized fall times of anisotropic samples significantly increase with increasing rate of magnetic-field change in comparison with isotropic samples (Figure 6b). For example, for an anisotropic sample with $\Gamma_{0}=0.6$ at 505 kA/(s·m), ${\tilde{t}}_{f}$ was approximately twice higher than for an isotropic sample. It seems that, if the magnetic-field rate is high, a pre-structured MAE cylinder needs more time to restore its original shape and internal microstructure than its isotropic (randomly heterogeneous) counterpart. This is an unexpected result in search of theoretical explanation.

**Figure 7:**
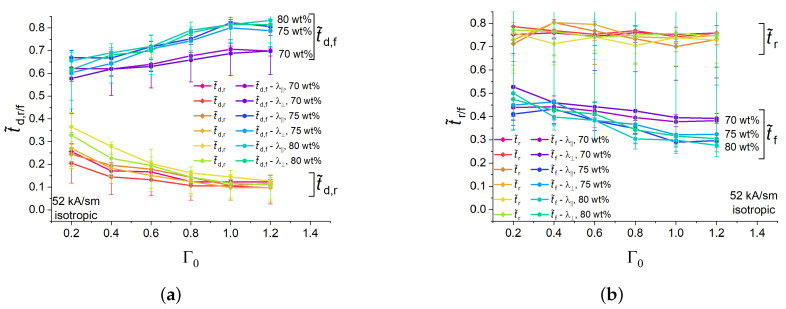
(**a**) Dependencies of normalised rising and falling delay times of isotropic MAE samples with different concentrations of Fe particles on the aspect ratio $\Gamma_{0}$ for longitudinal and transversal strains. (**b**) Dependencies of normalised rising and falling times of isotropic MAE samples with different concentrations of Fe particles on the aspect ratio $\Gamma_{0}$ for longitudinal and transversal strains.

Figure 7 serves to address the question if the characteristic times were dependent on the volume fraction of the soft magnetic filler and the direction of deformation (longitudinal ($\lambda_{\|}$) versus transversal ($\lambda_{⊥}$) strain). First, of all, the characteristic times for $\lambda_{\|}$ and $\lambda_{⊥}$ at otherwise the same experimental parameters were within the uncertainty of measurements. This is due to the higher uncertainty in measuring $\lambda_{⊥}$ as compared with $\lambda_{\|}$. The cylinder width was calculated as the distance between the experimentally determined left and right vertical cylinder edges. The magnitude of the apparent strain in the transverse direction was significantly lower (by a factor of roughly 0.3 [11]) than the strain magnitude in the longitudinal direction due to the transverse contraction and dent formation on the upper cylinder surface.

As far as the longitudinal strain was concerned, some differences in the transient behaviour of MAE samples with different iron concentrations were noticed. These differences are described in this passage below. The dependencies of ${\tilde{t}}_{d,f}$ on the aspect ratio $\Gamma_{0}$ were within the measurement uncertainty for the samples with 80 and 75 wt% of Fe, while, for the higher aspect ratios $\Gamma_{0}>0.4$, ${\tilde{t}}_{d,f}$ in less-filled samples with 70 wt% of Fe was significantly lower (about 0.1 at $\Gamma_{0}=1.2$) than in those. ${\tilde{t}}_{d,r}$ values for 80 and 75 wt% of Fe were practically indistinguishable from each other, while ${\tilde{t}}_{d,r}$ for 70 wt% of Fe was significantly higher than in higher-filled samples with small $\Gamma_{0}<0.6$. Within the uncertainty of measurement, the normalized rise time ${\tilde{t}}_{r}$ did not show any pronounced dependencies on $\Gamma_{0}$ or the weight fraction of the filler, ${\tilde{t}}_{r}\approx 0.75$. For higher aspect ratios $\Gamma_{0}>0.8$, ${\tilde{t}}_{f}$ in MAE samples containing 75 and 80 wt% of Fe were similar, and somewhat lower than ${\tilde{t}}_{f}$ in MAE samples with 70 wt% of iron (Figure 7b). For the two higher loaded materials, ${\tilde{t}}_{f}$ slightly decreased with increasing $\Gamma_{0}$ (${\tilde{t}}_{f}$ at $\Gamma_{0}$ = 1.2 is ≈0.1 smaller than at $\Gamma_{0}=0.2$.)

With the present state-of-the-art of research on magneto-mechanical interactions in MAEs, it is not possible either to explain or to predict the dependence of the transient behaviour of MAE cylinders on the volume fraction of the filler, due to the lack of suitable theoretical models.

### 3.2. Dependence on the Maximum Value of Magnetic Field

In the second set of experiments, we compared the characteristics times for different maximum values of magnetic field given in Table 2 at a fixed magnetic slew rate of 52 kA/(s·m) (current slew rate of 1 A/s). The time interval to reach the stationary state was increased from 20 s to 60 s because, from preliminary experiments, it was noticed that the transient processes for low values of $H_{\max}$ (hence, low maximum strains) may take more time, in particular for the power-down transition.

**Figure 8:**
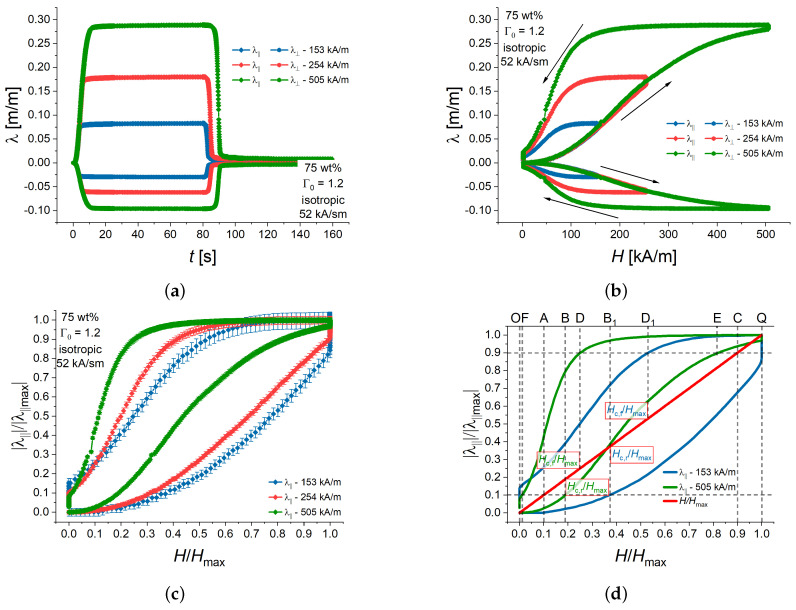
(**a**) Time dependencies of longitudinal and transversal strains of an isotropic MAE cylinder with 75 wt% of iron and $\Gamma_{0}=1.2$ for three different amplitudes of magnetic field at a fixed magnetic field rate 52 kA/(s·m). The error bars are omitted for clarity. (**b**) Longitudinal and transversal strains as functions of the momentary value of the magnetic field strength (magnetostrictive hysteresis loops). The error bars are omitted for clarity. (**c**) Normalized representation of the magnetostrictive hysteresis loops. (**d**) Schematic diagram of the determination of the normalized characteristic times and critical fields from a normalized hysteresis loop. The curves are smoothed by the running average algorithm.

Figure 8 shows measurement results for isotropic MAE cylinders with 75 wt% of iron particles and the aspect ratio of $\Gamma_{0}=1.2$. Figure 8a presents the time dependencies of the longitudinal and transverse strains. In Figure 8b, the same results are plotted as the magnetostrictive hysteresis loops. The shapes of hysteresis loops for different amplitudes of magnetic field were significantly different, which can be easily seen from the normalized hysteresis loops in Figure 8c, where both the longitudinal strain and the external magnetic fields are normalized on their maximum values. These normalized hysteresis loops allow one to deduce the normalized delay times ${\tilde{t}}_{d,r}$, ${\tilde{t}}_{d,f}$ from them directly (Figure 8d). It can be easily seen that $\left|AB\right|=0.8{\tilde{t}}_{d,r}$ and $\left|CD\right|=0.8{\tilde{t}}_{d,f}$. It is also directly visible that ${\tilde{t}}_{d,r}$ is higher for $H_{max}$ of 153 kA/m than for 505 kA/m, and ${\tilde{t}}_{d,f}$ is lower for $H_{max}$ of 153 kA/m than for 505 kA/m, because $\left|AB\right|<\left|AB_{1}\right|$ and $\left|CD\right|>\left|CD_{1}\right|$. To the best of our knowledge, such quantitative relationships between the characteristic response times and the hysteresis loop taken at dynamic conditions are proposed for the first time. The normalized rise and fall times ${\tilde{t}}_{r}$, ${\tilde{t}}_{f}$ can only be figured out from the magnetostrictive hysteresis loop if the corresponding events occurred before the magnetic field has reached its maximum or minimum value, respectively. In Figure 8d, this is indeed the case for the higher $H_{max}$ of 505 kA/m, where $\left|BE\right|=0.8{\tilde{t}}_{r}$ and $\left|DF\right|=0.8{\tilde{t}}_{f}$. It is not possible to determine ${\tilde{t}}_{r}$ and ${\tilde{t}}_{f}$ for $H_{max}$ of 153 kA/m from Figure 8d because of the strain creep. It can be only done from time measurements.

**Figure 9:**
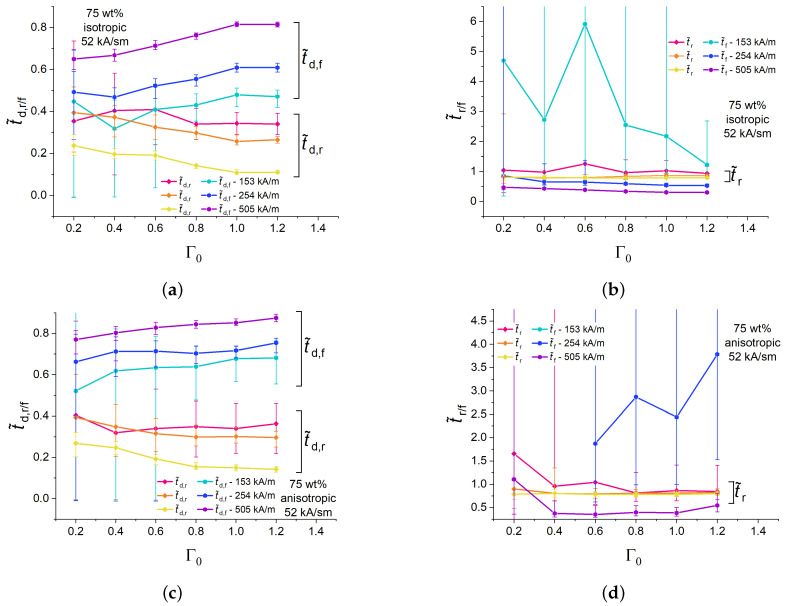
Dependencies of the normalized rise and fall delay times of isotropic (**a**) and anisotropic (**c**) MAE cylinders with 75 wt% of iron particles on the aspect ratio $\Gamma_{0}$ for different magnetic field amplitudes $H_{max}$ at a fixed value of the magnetic field rate of 52 kA/(s·m). Dependencies of the normalized rise and fall times of isotropic (**b**) and anisotropic (**d**) MAE cylinders with 75 wt% of iron particles on the aspect ratio $\Gamma_{0}$ for different magnetic field amplitudes $H_{max}$ at a fixed value of the magnetic field rate of 52 kA/(s·m).

The dependencies of the normalized characteristic times of isotropic and anisotropic MAE cylinders with 75 wt% of Fe on the aspect ratio $\Gamma_{0}$ for different amplitudes of magnetic field at a fixed magnetic field rate of 52 kA/(s·m) are shown in Figure 9. The experimental points in Figure 9 refer to the longitudinal strain. Qualitatively, the aspect-ratio dependencies of the normalized delay times (Figure 9a,c) were like those for the highest $H_{max}$ (Figure 5a,c). For specimens with sufficiently high aspect ratio $\Gamma_{0}>0.4$, ${\tilde{t}}_{d,r}$ tended to decrease with increasing magnetic field amplitude $H_{max}$, while ${\tilde{t}}_{d,f}$ tended to increase with increasing magnetic field amplitude $H_{max}$. The changes in the normalized delay times with varying $H_{max}$ originate from the changes in the shape of normalized magnetostrictive hysteresis loops as discussed above. For specimens with sufficiently high aspect ratio $\Gamma_{0}>0.2$, both ${\tilde{t}}_{r}$ and ${\tilde{t}}_{f}$ tended to increase with decreasing magnetic field amplitude $H_{max}$.

In Figure 9d, some points for ${\tilde{t}}_{f}$ are not available because the lower reference level (10%) of the maximum strain was not reached during the measurement. An example of such a situation is shown in Appendix C. This effect was observed only for anisotropic samples and low magnetic field amplitudes $H_{max}$, which can be attributed to the previously reported remanent strain [6,11].

Large error bars are clearly visible in Figure 9. They originate from small changes in the cylinder height, where the pixel size becomes comparable with an elongation Δh. Obviously, low values of Δh are observed at low $\Gamma_{0}$ and/or low $H_{max}$.

## 4. Conclusions

We have presented a detailed investigation of strain dynamics for MAE cylinders with different concentrations and arrangements (initially randomly heterogeneous and pre-structured materials) of iron particles in trapezoidal time-varying magnetic fields with four different slew rates of both legs and three different maximum values of magnetic field. The following main conclusions can be drawn:

- Introduced delay times of the magnetostrictive strain response for the increasing (ascending) and descending (decreasing) parts of magnetic fields were different in a particular experimental setting. For two higher magnetic field slew rates (505 kA/(s·m) and 757 kA/(s·m)) and otherwise the same experimental parameters, the delay time for the falling part of magnetic field was lower than the delay time for the rising magnetic field. Typically, ${\tilde{t}}_{d,r}\approx 0.2$, ${\tilde{t}}_{d,f}\approx 0.7$ for isotropic samples (Figure 5a).
- Introduced rise and fall times of the magnetostrictive strain response for the increasing (ascending) and descending (decreasing) parts of magnetic fields were different in a particular experimental setting. For isotropic samples and otherwise the same experimental parameters, the rise time for the ascending part of magnetic field was higher than the fall time for the descending magnetic field. Typically, ${\tilde{t}}_{r}\approx 0.8$, ${\tilde{t}}_{f}\approx 0.5$ for isotropic samples (Figure 6a).
- Characteristic times of anisotropic specimens were similar to those of their isotropic counterparts except for the falling time constant ${\tilde{t}}_{f}$, which was significantly higher in anisotropic samples than in isotropic samples (Figure 6b). This effect was particularly pronounced at the highest magnetic field rate of 757 kA/(s·m).
- At the same experimental conditions, the characteristic times of specimens with 80 and 75 wt% of iron were very close, while the characteristic times ${\tilde{t}}_{d,f}$, ${\tilde{t}}_{f}$ for the falling part of magnetic field of the specimen with 70 wt% of iron were different from those (Figure 7).
- A new graphical method for deducing the normalized delay times from the normalized magnetostrictive hysteresis curves has been presented (Figure 8d). The changes in the characteristic response times for different maximum values of magnetic field have been explained by the changing shape of the magnetostrictive hysteresis loop.
- At a fixed magnetic field rate of 52 kA/(s·m) and sufficiently high aspect ratio, ${\tilde{t}}_{d,r}$ increased with decreasing magnetic field amplitude $H_{max}$, while ${\tilde{t}}_{d,f}$ decreased. ${\tilde{t}}_{f}$ strongly decreased with increasing magnetic field amplitude $H_{max}$, while the decrease in ${\tilde{t}}_{r}$ was minor (Figure 9).

To the best of our knowledge, the transient behaviour of magnetostrictive strains of MAE cylinders has not been studied so far. We hope that the presented experimental study will encourage theoreticians to give quantitative explanations of features and effects observed. Further experimental research is required to better understand the deformation dynamics of MAE objects in time varying magnetic fields. In this context, the investigations of MAE ellipsoids could shed light on the underlying physics because, in this truly exceptional case, the magnetic field strength is uniform throughout the homogeneous body under the assumption of uniform, or zero, externally applied field [48], which may bring simplifications into theoretical considerations.

## Figures and Tables

**Table 1:** Driving current slew rates $\left|\frac{dI}{dt}\right|$ and corresponding slew rates $\left|\frac{dH}{dt}\right|$ of the external magnetic field.

$\left|\frac{\mathrm{dI}}{\mathrm{dt}}\right|$ [A/s]	$\left|\frac{\mathrm{dH}}{\mathrm{dt}}\right|$ [kA/(s·m)]
1	52 ± 1
5	254 ± 6
10	505 ± 13
15	757 ± 19

**Table 2:** Maximum current values $I_{max}$ of transient driving current and corresponding maximum values of magnetic field strength $H_{max}$.

$I_{\max}$ [A]	$H_{\max}$ [kA/m]
3	153
5	254
10	505

**Figure A1:**
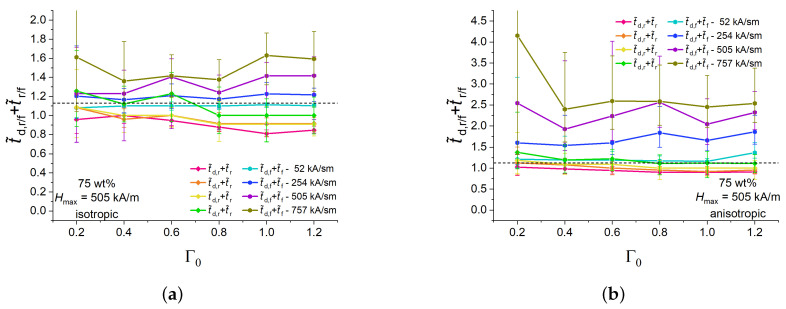
(**a**) Summary of Figure 5a and Figure 6a. (**b**) Summary of Figure 5c and Figure 6b. Dashed line corresponds to the numerical value of 1.125.

**Figure A2:**
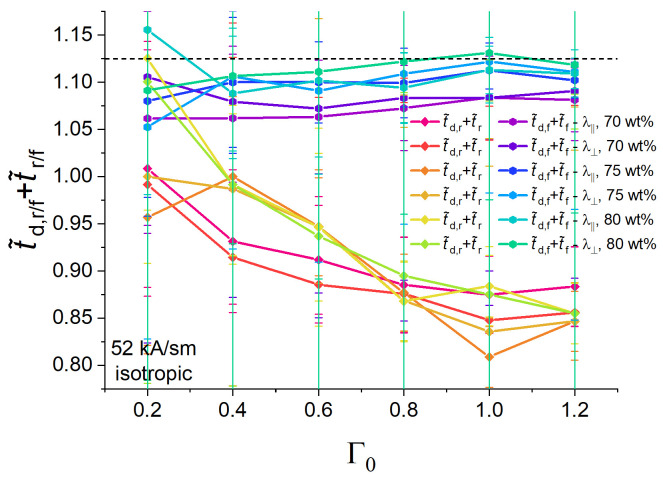
Summary of Figure 7a,b. Dashed line corresponds to the numerical value of 1.125.

**Figure A3:**
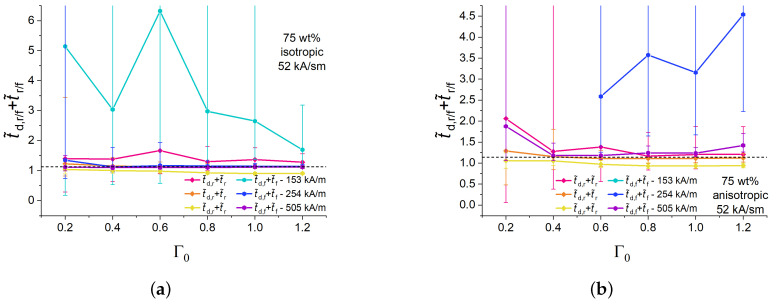
(**a**) Summary of Figure 9a,b. (**b**) Summary of Figure 9c,d. Dashed line corresponds to the numerical value of 1.125.

**Figure A4:**
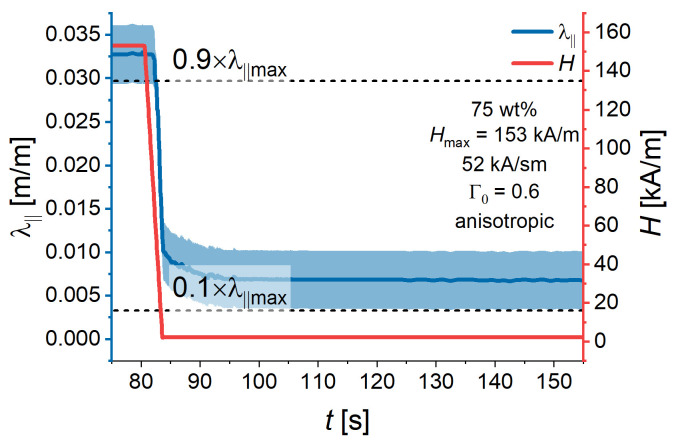
Measured time dependencies of the magnetic field strength (red line) and the longitudinal strain (blue line) for an anisotropic sample with 75% of iron and $\Gamma_{0}=0.6$. The blue area designates the measurement uncertainty. The dashed lines stand for the reference values. Other experimental parameters are given in the figure.

## Data Availability

The data supporting the findings of this paper are available in Zenodo: https://doi.org/10.5281/zenodo.10679499 (accessed on 14 February 2024).

## Appendix A

Let us derive Equations (3) and (4). From Figure 8d, we obtain $\left|OA\right|+\left|AB\right|=H_{c,r}/H_{max}$, where |OA|=0.1 and $\left|AB\right|=0.8{\tilde{t}}_{d,r}$. The multiplier 0.8 comes from the normalization on the rise time of the ramp (0.9−0.1=0.8). Similarly, for a negative-going transition, one gets $\left|QC\right|+\left|CD\right|=\left[1-(H_{c,f}/H_{max})\right]$, where |QC|=0.1 and $\left|CD\right|=0.8{\tilde{t}}_{d,f}$.

## Appendix B

In this Appendix, we present the sum of the normalized characteristics times $\left({\tilde{t}}_{d,r}+{\tilde{t}}_{r}\right)$ and $\left({\tilde{t}}_{d,f}+{\tilde{t}}_{f}\right)$ for all the experimental results shown in the main body of this paper.

## Appendix C

An example is given when the time $t_{f}$ was not determined from the measurement. It is visible that the blue line does not cross the lower reference value.

## References

[1] Elhajjar R., Law C.T., Pegoretti A. (2018). Magnetostrictive polymer composites: Recent advances in materials, structures and properties. Prog. Mater. Sci..

[2] Romeis D., Toshchevikov V., Saphiannikova M. (2019). Effects of local rearrangement of magnetic particles on deformation in magneto-sensitive elastomers. Soft Matter.

[3] Stolbov O.V., Raikher Y.L. (2019). Magnetostriction effect in soft magnetic elastomers. Arch. Appl. Mech..

[4] Sánchez P.A., Stolbov O.V., Kantorovich S.S., Raikher Y.L. (2019). Modeling the magnetostriction effect in elastomers with magnetically soft and hard particles. Soft Matter.

[5] Kalina K.A., Metsch P., Brummund J., Kästner M. (2020). A macroscopic model for magnetorheological elastomers based on microscopic simulations. Int. J. Solids Struct..

[6] Saveliev D.V., Belyaeva I.A., Chashin D.V., Fetisov L.Y., Romeis D., Kettl W., Kramarenko E.Y., Saphiannikova M., Stepanov G.V., Shamonin M. (2020). Giant Extensional Strain of Magnetoactive Elastomeric Cylinders in Uniform Magnetic Fields. Materials.

[7] Bastola A.K., Hossain M. (2021). The shape—Morphing performance of magnetoactive soft materials. Mater. Des..

[8] Silva J., Gouveia C., Dinis G., Pinto A., Pereira A. (2022). Giant magnetostriction in low-concentration magnetorheological elastomers. Compos. Part B Eng..

[9] Balogh D., Guba S., Horváth B., Szalai I. (2022). Magnetic Field-Induced Deformation of Isotropic Magnetorheological Elastomers. Magnetochemistry.

[10] Tasin M.A., Aziz S.A.A., Mazlan S.A., Johari M.A.F., Nordin N.A., Yusuf S.Y.M., Choi S.B., Bahiuddin I. (2023). Magnetostriction Enhancement in Midrange Modulus Magnetorheological Elastomers for Sensor Applications. Micromachines.

[11] Glavan G., Belyaeva I.A., Drevenšek-Olenik I., Shamonin M. (2023). Experimental study of longitudinal, transverse and volume strains of magnetoactive elastomeric cylinders in uniform magnetic fields. J. Magn. Magn. Mater..

[12] Roghani M., Romeis D., Saphiannikova M. (2023). Effect of microstructure evolution on the mechanical behavior of magneto-active elastomers with different matrix stiffness. Soft Matter.

[13] Goh S., Menzel A.M., Wittmann R., Löwen H. (2023). Density functional approach to elastic properties of three-dimensional dipole-spring models for magnetic gels. J. Chem. Phys..

[14] Ubaidillah, Sutrisno J., Purwanto A., Mazlan S.A. (2015). Recent Progress on Magnetorheological Solids: Materials, Fabrication, Testing, and Applications. Adv. Eng. Mater..

[15] Menzel A.M. (2015). Tuned, driven, and active soft matter. Phys. Rep..

[16] Lopez-Lopez M.T., Durán J.D.G., Iskakova L.Y., Zubarev A.Y. (2016). Mechanics of Magnetopolymer Composites: A Review. J. Nanofluids.

[17] Weeber R., Hermes M., Schmidt A.M., Holm C. (2018). Polymer architecture of magnetic gels: A review. J. Phys. Condens. Matter.

[18] Bastola A.K., Paudel M., Li L., Li W. (2020). Recent progress of magnetorheological elastomers: A review. Smart Mater. Struct..

[19] Odenbach S. (2021). Magnetic Hybrid-Materials: Multi-Scale Modelling, Synthesis, and Applications.

[20] Nadzharyan T.A., Shamonin M., Kramarenko E.Y. (2022). Theoretical Modeling of Magnetoactive Elastomers on Different Scales: A State-of-the-Art Review. Polymers.

[21] Kostrov S.A., Marshall J.H., Maw M., Sheiko S.S., Kramarenko E.Y. (2023). Programming and Reprogramming the Viscoelasticity and Magnetic Response of Magnetoactive Thermoplastic Elastomers. Polymers.

[22] Domenjoud M., Berthelot E., Galopin N., Corcolle R., Bernard Y., Daniel L. (2019). Characterization of giant magnetostrictive materials under static stress: Influence of loading boundary conditions. Smart Mater. Struct..

[23] Boyraz P., Runge G., Raatz A. (2018). An Overview of Novel Actuators for Soft Robotics. Actuators.

[24] Bernat J., Gajewski P., Kołota J., Marcinkowska A. (2023). Review of Soft Actuators Controlled with Electrical Stimuli: IPMC, DEAP, and MRE. Appl. Sci..

[25] Buschow K.H.J., Boer F.R. (2003). Physics of Magnetism and Magnetic Materials.

[26] Ivaneyko D., Toshchevikov V., Saphiannikova M., Heinrich G. (2014). Mechanical properties of magneto-sensitive elastomers: Unification of the continuum-mechanics and microscopic theoretical approaches. Soft Matter.

[27] Dapino M.J. (2002). Magnetostrictive Materials. Encyclopedia of Smart Materials.

[28] Belyaeva I.A., Kramarenko E.Y., Stepanov G.V., Sorokin V.V., Stadler D., Shamonin M. (2016). Transient magnetorheological response of magnetoactive elastomers to step and pyramid excitations. Soft Matter.

[29] Wen Q., Wang Y., Feng J., Gong X. (2018). Transient response of magnetorheological elastomers to step magnetic field. Appl. Phys. Lett..

[30] Selzer L., Odenbach S. (2023). Empirical Law for the Magnetorheological Effect of Nanocomposite Hydrogels with Magnetite Microparticles. Gels.

[31] Selzer L., Odenbach S. (2023). Mechanism for the Magnetorheological Effect of Nanocomposite Hydrogels with Magnetite Microparticles. Gels.

[32] Belyaeva I.A., Kramarenko E.Y., Shamonin M. (2017). Magnetodielectric effect in magnetoactive elastomers: Transient response and hysteresis. Polymer.

[33] Kubík M., Borin D., Odenbach S. (2023). Transient dynamics of the field induced force in the isotropic magnetorheological elastomer. Smart Mater. Struct..

[34] Johnson B.K., Sundaram V., Naris M., Acome E., Ly K., Correll N., Keplinger C., Humbert J.S., Rentschler M.E. (2020). Identification and Control of a Nonlinear Soft Actuator and Sensor System. IEEE Robot. Autom. Lett..

[35] Rothemund P., Kirkman S., Keplinger C. (2020). Dynamics of electrohydraulic soft actuators. Proc. Natl. Acad. Sci. USA.

[36] Mazurek P., Vudayagiri S., Skov A.L. (2019). How to tailor flexible silicone elastomers with mechanical integrity: A tutorial review. Chem. Soc. Rev..

[37] Sorokin V.V., Belyaeva I.A., Shamonin M., Kramarenko E.Y. (2017). Magnetorheological response of highly filled magnetoactive elastomers from perspective of mechanical energy density: Fractal aggregates above the nanometer scale?. Phys. Rev. E.

[38] Glavan G., Belyaeva I.A., Ruwisch K., Wollschläger J., Shamonin M. (2021). Magnetoelectric Response of Laminated Cantilevers Comprising a Magnetoactive Elastomer and a Piezoelectric Polymer, in Pulsed Uniform Magnetic Fields. Sensors.

[39] Horváth B., Szalai I. (2023). Magnetic susceptibility and response time of isotropic and structured magnetorheological elastomers. J. Intell. Mater. Syst. Struct..

[40] Kubík M., Válek J., Žáček J., Jeniš F., Borin D., Strecker Z., Mazůrek I. (2022). Transient response of magnetorheological fluid on rapid change of magnetic field in shear mode. Sci. Rep..

[41] Szewczyk R. (2019). Model of the Magnetostrictive Hysteresis Loop with Local Maximum. Materials.

[42] Wan Y., Fang D., Hwang K.C. (2003). Non-linear constitutive relations for magnetostrictive materials. Int. J. -Non-Linear Mech..

[43] (2011). IEEE Standard for Transitions, Pulses, and Related Waveforms (Revision of IEEE Std 181-2003).

[44] Engelberg S. (2005). A Mathematical Introduction to Control Theory.

[45] Zubarev A., Chirikov D., Stepanov G., Borin D., Lopez-Lopez M. (2018). On the theory of hysteretic magnetostriction of soft ferrogels. Phys. A Stat. Mech. Its Appl..

[46] Belyaeva I.A., Klepp J., Lemmel H., Shamonin M. (2021). Feasibility of Probing the Filler Restructuring in Magnetoactive Elastomers by Ultra-Small-Angle Neutron Scattering. Appl. Sci..

[47] Chen K., Watanabe M., Takeda Y., Maruyama T., Uesugi M., Takeuchi A., Suzuki M., Uesugi K., Yasutake M., Kawai M. (2022). In situ observation of the movement of magnetic particles in polyurethane elastomer Densely Packed Magnetic Particles Using Synchrotron Radiation X-ray Computed Tomography. Langmuir.

[48] Beleggia M.D.G.M., Millev Y. (2006). Demagnetization factors of the general ellipsoid: An alternative to the Maxwell approach. Philos. Mag..

